# Acute Intestinal Obstruction Complicating Abdominal Pregnancy: Conservative Management and Successful Outcome

**DOI:** 10.1155/2016/2576280

**Published:** 2016-05-26

**Authors:** Gerald Okanandu Udigwe, George Uchenna Eleje, Eric Chukwudi Ihekwoaba, Onyebuchi Izuchukwu Udegbunam, Richard Obinwanne Egeonu, Ayodele Obianuju Okwuosa

**Affiliations:** ^1^Department of Obstetrics and Gynecology, Faculty of Medicine, College of Health Sciences, Nnamdi Azikiwe University, Nnewi Campus, PMB 5001, Nnewi, Anambra, Nigeria; ^2^Department of Obstetrics and Gynecology, Nnamdi Azikiwe University Teaching Hospital, PMB 5025, Nnewi, Anambra, Nigeria; ^3^Department of Surgery, Nnamdi Azikiwe University Teaching Hospital, PMB 5025, Nnewi, Anambra, Nigeria

## Abstract

*Background*. Acute intestinal obstruction during pregnancy is a very challenging and unusual nonobstetric surgical entity often linked with considerable fetomaternal morbidity and mortality. When it is synchronous with abdominal pregnancy, it is even rarer.* Case Presentation*. A 28-year-old lady in her second pregnancy was referred to Nnamdi Azikiwe University Teaching Hospital, Nnewi, Nigeria, at 27 weeks of gestation due to vomiting, constipation, and abdominal pain. Examination and ultrasound scan revealed a single live intra-abdominal extrauterine fetus. Plain abdominal X-ray was diagnostic of intestinal obstruction. Conservative treatment was successful till the 34-week gestational age when she had exploratory laparotomy. At surgery, the amniotic sac was intact and the placenta was found to be adherent to the gut. There was also a live female baby with birth weight of 2.3 kg and Apgar scores of 9 and 10 in the 1st and 5th minutes, respectively, with the baby having right clubbed foot. Adhesiolysis and right adnexectomy were done. The mother and her baby were well and were discharged home nine days postoperatively.* Conclusion*. To the best of our knowledge, this is the first report of abdominal pregnancy as the cause of acute intestinal obstruction in the published literature. Management approach is multidisciplinary.

## 1. Introduction

The occurrence of acute intestinal obstruction as a cause of acute abdominal pain during pregnancy is a rare entity. For instance, the incidence of intestinal obstruction complicating pregnancy ranges from 1 in 1,500 to 1 in 66,431 deliveries [1]. When it is synchronous with abdominal pregnancy, it is rarer and often heralded with a mammoth of diagnostic and therapeutic challenges. When diagnosis is missed or delayed, it could lead to intestinal strangulation, which results in a high incidence of maternal morbidity, mortality, and fetal loss [2, 3]. Reduction in the incidence of these complications largely depends on early detection of the problem but, unfortunately, accurate diagnosis is often difficult in pregnancy [1]. To the best of our knowledge, this is the first case of acute intestinal obstruction complicating abdominal pregnancy with successful outcome.

## 2. Case Presentation

A 28-year-old lady, at her second pregnancy, but without any living child, came to our facility on referral from a private hospital (about 19 miles from our facility). She presented to the accident and emergency department at 27-week gestational age, on account of vomiting, constipation, and abdominal pain of 3-day duration prior to presentation. She had no previous history of abdominopelvic surgery. She came with a referral note, stating that she was being managed for acute intestinal obstruction in pregnancy to rule out red degeneration of fibroids.

Examination revealed a young lady in painful distress. She was not febrile, not pale, anicteric, acyanosed, and with no pedal edema. A nasogastric tube was in situ draining bilious effluent. Her temperature was 37.2°C, respiratory rate was 26 cycles per minute, pulse rate was 102 beats/minute, and blood pressure was 120/70 mmHg.

The abdomen was enlarged and moved with respiration. There was generalized mild tenderness. The fetal parts were easily palpable. The bowel sounds were present and hyperactive. Hemoglobin was 10 g/dL, serum electrolytes were within normal range, and urine analysis showed no abnormal findings. The blood group was O rhesus D positive and hemoglobin genotype was AA.

An emergency ultrasound scan revealed a single live intra-abdominal extrauterine fetus in transverse lie with fetal head and trunk lying just deep to the anterior abdominal wall with estimated gestational age of 27 weeks. Plain abdominal X-ray was diagnostic of intestinal obstruction. The diagnosis of acute intestinal obstruction secondary to abdominal pregnancy was made. She was comanaged by the obstetrics team and the general surgery team. Subsequently, she was placed on nil per oral, while a nasogastric tube was left in place to decompress the abdomen. She was also placed on intravenous omeprazole 80 mg stat and then 40 mg twice daily, intravenous amoxicillin-clavulanic acid 1.2 g 12-hourly, intravenous metronidazole 500 mg 8-hourly, and analgesics and intravenous fluid of 5% dextrose to be alternated with normal saline. Strict fluid input and output were maintained. Four units of blood were grouped, cross-matched, and kept in the blood bank. There was complete return of bowel function after the initial management. Following resolution of symptoms, she was transferred from the accident and emergency department to the prenatal ward of the hospital for conservative management.

She was commenced on graded oral feeds on day seven of admission. Close fetomaternal monitoring was instituted. She received 24 mg of dexamethasone in 2 divided doses to enhance fetal lung maturation.

Following admission, she remained stable till the 33rd day on admission at gestational age of 32 weeks and 2 days when she developed signs of repeat intestinal obstruction which resolved within 48 hours. She was booked for an elective abdominal delivery/exploratory laparotomy at the 34th week of gestation. The comanaging General Surgery Unit was notified and invited in view of the abdominal placentation and recurrent history of intestinal obstruction. The neonatology team was also in attendance.

**Figure 1:**
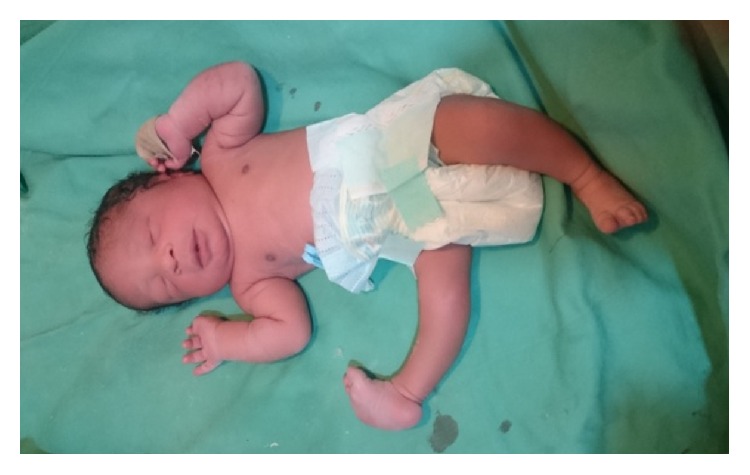
The baby following delivery.

A midline subumbilical incision was made under general anesthesia with endotracheal intubation. Intraoperative findings included intact amniotic sac and fetus that lied on gut where the placenta had its attachment and no other cause of intestinal obstruction was found. The uterus had fundal subserosal fibroid that measured 4 cm by 4 cm and a live female baby that weighed 2.3 kg with Apgar scores of 9 and 10 in the 1st and 5th minutes, respectively, with right clubbed foot, facial asymmetry, clitoromegaly, and hypertrophy of the labia majora (Figure 1). The placenta weighed 1.2 kg. The adherent gut on the placenta was freed through adhesiolysis, and right adnexectomy was done (right salpingo-oophorectomy and excision of the right broad ligament). Estimated blood loss was one litre. She received two units of whole blood intraoperatively.

Following the surgery, she was observed at the recovery room and later transferred to the lying-in ward. The baby was kept at the special care baby unit following the delivery, where she was evaluated and discharged to the mother on the 7th day of birth.

Recovery was uneventful and postoperative hematocrit was 27%. She produced an adequate amount of urine throughout this period. She had alternate sutures removed on the 8th day postoperatively. On the 9th day, the remaining sutures were removed and the patient was discharged home on oral hematinics and antibiotics. The baby was referred to the orthopedic surgery team for correction of the foot deformity.

## 3. Discussion

Although acute intestinal obstruction during pregnancy is a rare entity, maternal morbidity and mortality, as well as fetal loss, are usually high [1–3]. A number of conditions could predispose to occurrence of intestinal obstruction in pregnancy. In one previous report in the published literature, Connolly et al. reported that the common causes of intestinal obstruction in pregnancy were adhesions (54.6%), volvulus not due to adhesions (24.5%), intussusception (5.1%), carcinoma (3.7%), hernia (1.4%), and others (10.7%) [2]. Our patient had no previous history of abdominopelvic surgery. Our team has become the first to report abdominal pregnancy as the cause of intestinal obstruction in the published literature, to the best of our knowledge.

When abdominal pregnancy on its own entity is considered, the possible risk factors for it include tubal damage, pelvic inflammatory disease, endometriosis, multiparity, and in vitro fertilization [4]. The patient under review had 9 years of secondary infertility. In the previous cases of abdominal pregnancy in our hospital, there were also histories of infertility [5, 6].

The possible sites of abdominal pregnancy include the omentum, pelvic sidewall, broad ligament, posterior cul-de-sac, abdominal organs (spleen, liver, bowel, etc.), pelvic vessels, and diaphragm [7–9]. Luckily for our patient, the right adnexum was the site of implantation with some part of the placenta adherent to gut and despite these attachments of the placenta it was not so difficult to handle intraoperatively.

It is unclear whether abdominal pregnancy is a result of secondary implantation from an aborted tubal pregnancy or the result of intra-abdominal fertilization of sperm and ovum with primary implantation in the abdomen [10]. It is possible that the patient under review had secondary abdominal pregnancy from aborted right tubal ectopic pregnancy as the right tube was damaged as seen intraoperatively.

Due to variable location of abdominal pregnancy, it is associated with a wide range of symptoms and signs. In contrast to tubal ectopic pregnancy, it may not be detected till advanced gestation. Our patient presented as a result of intestinal obstruction at a gestational age of 27 weeks. Abdominal pain is a common feature, but the displacement of abdominal organs as pregnancy progresses results in atypical location of the pain and hence could delay diagnosis. This case is unique from others previously managed in our hospital because, unlike in others, the diagnosis was made during the antenatal period [5, 6]. Since she presented with features of intestinal obstruction, this could have been responsible for its accurate diagnosis in the antenatal period because of the raised suspicion.

Additionally, our patient had vaginal bleeding at early gestation and this could have also raised the suspicion of ectopic pregnancy. This is not surprising as the endometrium still responds to changes in pregnancy [11]. Some patients may present with an acute abdominal pain and shock due to severe hemorrhage from placental separation or rupture of viscus or maternal blood vessels [11, 12]. In undiagnosed cases, others may present as a result of failed induction due to lack of myometrial response to oxytocin and/or inability to ripen the cervix with intracervical Foley catheter and misoprostol tablet [13].

For acute diagnosis of abdominal pregnancy, high index of suspicion is necessary. The ultrasound showed the absence of myometrial tissue between the maternal bladder and the fetal parts [10]. Our patient had a similar ultrasound finding. However, it is important to note that the referral hospital could not make the diagnosis. Diagnosis of abdominal pregnancy was only made at presentation in our hospital. An advanced abdominal pregnancy may be misinterpreted as being intrauterine if the sonographer does not evaluate the myometrium during the scanning. Computerized tomographic scan or magnetic resonant imaging can be useful for confirming the diagnosis, distinguishing anatomic relationship and potential vascular connections, and assessing placenta adherence [14]. Although our patient did not do CT scan or MRI, she did Doppler ultrasound scan.

The treatment of abdominal pregnancy varies. If the diagnosis is made in early gestation, operative laparoscopy is an option [15]. If it is implanted on a vascular surface, due to the risk of hemorrhage, laparoscopy should be avoided. Methotrexate therapy has little effect on abdominal pregnancy [16]. However, if the diagnosis is made late in pregnancy, termination of pregnancy is an option since the possibility of delivery of a healthy infant is poor and the risk of maternal complication is high. A viable infant may be delivered via laparotomy. Our patient was delivered via laparotomy. Expectant management to gain fetal maturity has been attempted and has been successful in a few cases [17]. This was the option chosen for our patient as she had 9 years of secondary infertility.

The main treatment of advanced abdominal pregnancy is surgery. The main issue is how to manage the placenta. Ligation of the umbilical cord and leaving the placenta in situ are preferred by many due to the life-threatening maternal hemorrhage that may follow placenta removal. The patient can be monitored closely with no further treatment or with active intervention using arterial embolization or methotrexate can be instituted to accelerate involution [10, 18]. An alternative approach is to ligate the placental blood supply and then try to remove the placenta [10, 11]. This is mainly done if the placenta is not attached to a major structure. Our patient's placenta was attached to the right adnexum and was successfully removed.

Common abnormalities in infants that are the product of abdominal pregnancies include facial/or cranial asymmetry, joint abnormalities, hypoplastic limbs, and central nervous system abnormalities. The baby in the current case report had slight asymmetry, club foot, and clitoromegaly with hypertrophy of the labia majora. Generally, following abdominal pregnancy, the live birth rate is estimated at 10–20% while congenital anomalies occur in up to 40% of cases with about half of these surviving through the 1st seven days of life [3, 9].

In conclusion, to the best of our knowledge, this has become the first report of abdominal pregnancy as the cause of intestinal obstruction in the published literature. Acute intestinal obstruction complicating abdominal pregnancy with resultant healthy newborn is a rarity. Diagnosis and management of the condition can be very difficult and challenging and may need multidisciplinary approach. Intractable hemorrhage is the single most important life-threatening complication for the mother while fetal malformation is one of the numerous challenges that can confront the newborn.
